# Role of PA2G4P4 pseudogene in bladder cancer tumorigenesis

**DOI:** 10.3390/biology9040066

**Published:** 2020-03-31

**Authors:** Laura Pisapia, Sara Terreri, Pasquale Barba, Marianna Mastroianni, Maria Donnini, Vincenzo Mercadante, Alessandro Palmieri, Paolo Verze, Vincenzo Mirone, Vincenzo Altieri, Gianluigi Califano, Giovanna Lucia Liguori, Maria Strazzullo, Amelia Cimmino, Giovanna Del Pozzo

**Affiliations:** 1 Institute of Genetics and Biophysics, CNR, 80131 Naples, Italy; laura.pisapia@virgilio.it (L.P.); sara.terreri87@gmail.com (S.T.); pasquale.barba@igb.cnr.it (P.B.); marian.mastroianni@hotmail.com (M.M.); donnini.maria@libero.it (M.D.); vincenzo.mercadante@igb.cnr.it (V.M.); amelia.cimmino@igb.cnr.it (A.C.); 2 B Cell Pathophysiology Unit, IRCCS Bambino Gesù Children’s Hospital, 00146 Rome, Italy; 3 Department of Neurosciences, Reproductive Sciences and Odontostomatology, University of Naples Federico II, 80131 Naples, Italy; info@alessandropalmieri.it (A.P.); vincenzo.mirone@unina.it (V.M.); califano@unina.it (G.C.); 4 Department of Medicine and Surgery “Scuola medica Salernitana” University of Salerno, 84084 Salerno, Italy; Pverze@unisa.it (P.V.); valtieri@unisa.it (V.A.)

**Keywords:** oncogene, noncoding RNA, expression, EBP1, bladder cancer

## Abstract

Background: Many pseudogenes possess biological activities and play important roles in the pathogenesis of various types of cancer including bladder cancer (BlCa), which still lacks suitable molecular biomarkers. Recently, pseudogenes were found to be significantly enriched in a pan-cancer classification based on the Cancer Genome Atlas gene expression data. Among them, the top-ranking pseudogene was the proliferation-associated 2G4 pseudogene 4 *(PA2G4P4*). Methods: Genomic and transcript features of *PA2G4P4* were determined by GeneBank database analysis followed by 5’ RACE experiments. Therefore, we conducted a retrospective molecular study on a cohort of 45 patients of BlCa. *PA2G4P4* expression was measured by RT-qPCR, whereas *PA2G4P4* transcript distribution was analyzed by in situ hybridization on both normal and cancerous histological sections and compared to the immunolocalization of its parental *PA2G4/EBP1* protein. Finally, we tested the effects of *PA2G4P4* depletion on proliferation, migration, and death of BlCa cells. Results: We showed for the first time *PA2G4P4* overexpression in BlCa tissues and in cell lines. *PA2G4P4* distribution strictly overlaps PA2G4/EBP1 protein localization. Moreover, we showed that *PA2G4P4* knockdown affects both proliferation and migration of BlCa cells, highlighting its potential oncogenic role. Conclusions: *PA2G4P4* may play a functional role as an oncogene in BlCa development, suggesting it as a good candidate for future investigation and new clinical applications.

## 1. Introduction

Pseudogenes are evolutionally conserved and present in several organisms [1]. They derive from a retro-transposition event of a processed gene or from the degeneration of a duplicated gene. Over the last decades, data from large transcriptomic studies have shown that most pseudogenes are not simply “genomic relicts”, but are transcriptionally active and involved in important regulatory mechanisms [1]. Pseudogenes may act as positive or negative regulators of gene expression. As positive regulators, pseudogenes may compete with the parental genes for the same microRNAs or same destabilizing RNA-binding proteins, resulting in an increased level of the parental gene. As negative regulators, pseudogenes may compete for the same stabilizing RNA binding proteins or transcribe into endogenous siRNAs that can bind to any region of the parental genes [2,3]. 

Human genome encodes thousands of pseudogenes, many of which are differentially expressed in human cancers and in their normal tissue [4]. Pseudogenes have therefore attracted considerable attention as a new class of biomarkers that could provide new tumor-grade and -stage specific diagnostic and prognostic tools. 

Bladder cancer (BlCa) is the second most frequent genito-urinary malignancy and the sixth most common malignancy in men in the Western world [5]. To date, the only methods available for the diagnosis of BlCa are cystoscopy and urinary cytology. Cystoscopy is an invasive clinical procedure able to detect visible tumors irrespective of the histological grade, while cytology detects high-grade malignancy or carcinoma in situ (CIS). Generally, these two tests complement each other. Therefore, the identification of new molecular biomarkers for the early detection of BlCa or prediction of tumor recurrence and progression [6,7,8] is still a compelling request.

Recently, a comprehensive genomic pan-cancer classification, based on the Cancer Genome Atlas dataset analysis, identified a specific set of genes that could correctly classify most of the samples from several different kinds of cancer. Of note, one third of the top genes identified were pseudogenes, among which the most frequent was the proliferation-associated 2G4 pseudogene 4 *(PA2G4P4)* [9]. Its functional counterpart is the *PA2G4* gene, located on chromosome 12 and encoding the ErbB3-binding protein 1 or EBP1 [10,11]. EBP1 is a DNA- and RNA-binding protein ubiquitously expressed and involved in both physiological and pathological processes including tumorigenesis [12,13]. The *PA2G4P4* locus is located on chromosome 3 and included in the long intergenic non-coding RNA 886 (LINC00886). Both *PA2G4P4* and its functional counterpart *PA2G4* are expressed in BlCa, even though their role is still unclear [14]. In the present study, the structure, expression, and functional features of *PA2G4P4* in BlCa were analyzed for the first time, with the aim of investigating its potential value as a biomarker and therapeutic target in bladder tumorigenesis. 

## 2. Materials and Methods

*Cell lines*. Human J82 and RT112 cell lines were provided by American Type Culture Collection (Manassas, VA, USA). J82 were propagated and maintained as a monolayer in MEM (Merck KGaA, Darmstadt, Germany) and RT112 were grown in RPMI (Thermo Fisher Scientific, Inc., Waltham, MA, USA). Both cell lines were supplemented with 10% fetal bovine serum and 1% antibiotic and antimitotic mix (Merck KGaA). When they reached 80% confluence, they were diluted with 0.05% trypsin, and then cultured in 5% CO_2_ at 37 °C. 

*Patient samples.* Bladder biopsy samples (n = 79) were used to evaluate the expression of the PA2G4P4 pseudogene: 34 samples from non-pathological tissues termed normal bladder epithelium (NBE) and 45 samples from tumor tissues termed BlCa. All BlCa patients were classified according to the 2016 World Health Organization classification system (Table 1)

All subjects gave their informed consent for inclusion before they participated in the study. The study was conducted in accordance with the Declaration of Helsinki, and the protocol was approved by the Ethics Committee of Federico II University (245/19). Bladder tissue samples were harvested during transurethral bladder tumor resection performed at the Urology Unit of the University of Naples Federico II (Naples, Italy) from 2017 to 2019. Tumor tissue samples were obtained directly from the tumor site, whereas normal tissue samples were obtained from other sites of the bladder, which had no evidence of macroscopic alterations. 

Each tumor sample and normal-looked mucosa were histologically evaluated by two independent pathologists. All samples were stored in liquid nitrogen immediately following resection and transferred to −80 °C.

*5′ RACE of PA2G4P4 transcript.* To identify the 5′ end of the *PA2G4P4* transcript, total RNA was extracted from J82 cells using TRIzol and then DNase I (RNase-free; Thermo Fisher Scientific, Inc.), and the 5′/3′ RACE Kit, 2nd Generation (Roche Applied Science, Penzberg, Germany) was used to generate primer-specific cDNA, according to the manufacturer’s instructions. Three different gene-specific primers (Table 2) were designed in reverse, as compared to the *PA2G4P4* sequence. These primers covered 250 bp in the 5′ region of the annotated *PA2G4P4* sequence. The most internal primer (R1) was used to generate the primer-specific cDNA; the second primer (R2, partially overlapping with R1) was used to obtain the specific amplification; the third, more external primer (R3) was used to perform a semi-nested amplification. Through this approach, an elongation of 102 bp was obtained. The sample resulting from semi-nested amplification was purified, cloned in a TA cloning vector (TA Cloning® Kit; Thermo Fisher Scientific, Inc.), and sequenced. 

*RNA extraction and reverse transcription-quantitative polymerase chain reaction (RT-qPCR).* Total RNA was extracted from ~30 mg of BlCa and NBE tissues, and from 1 × 10^6^ J82 and RT112 BlCa cells using TRIzol reagent. The integrity of the RNA was checked using gel electrophoresis. Total RNA (1 μg) was reverse-transcribed using a QuantiTect Reverse Transcription Kit (Qiagen). RT-qPCR was performed using strand-specific primers and U6 small nuclear RNA was used as the reference gene [15] (Table 2). According to the manufacturer’s instructions, RT-qPCR analysis was performed using iQ SYBR Green Supermix (Bio-Rad Laboratories, Inc., Hercules, CA, USA) and the protocol for CFX96 Deep Well Real-Time PCR Detection System (Bio-Rad Laboratories, Inc.).

Each sample was analyzed in triplicate. The 2^-ΔΔct^ method was used for relative quantification of the gene expression, and the results are expressed as log_2_ (2^-ΔΔct^) [16].

*Histological analysis*. Tumor samples were fixed in 10% formalin for 24 h and then dehydrated and embedded in paraffin. Samples were then sectioned with a microtome to undergo various analyses. For microscopic examination, the paraffin-embedded sections were deparaffinized, rehydrated, and stained with hematoxylin-eosin [17].

*In situ hybridization of paraffin sections.* In situ hybridization on tissue sections was performed as previously described [15,18,19]. The *PA2G4P4* fragment (450 bp) was obtained by amplifying the non-*PA2G4* homolog region with the primers listed in Table 2. The obtained fragment was cloned in TOPO TA pCR2.1 cloning vector (Thermo Fisher Scientific, Inc.), according to the manufacturer’s instructions, and RNA antisense probes were synthesized from linearized plasmid in the presence of Digoxigenin-UTP (Roche Diagnostics GmbH, Mannheim, Germany). Tissue sections were mounted directly or after Fast Red staining. Stained sections were examined and photographed using a Leica MZ12 dissection and a Nikon ECLIPSE Ni microscope (Nikon Corporation, Tokyo, Japan). All images were processed in Adobe Photoshop 10.0 (Adobe Systems, Inc., San Jose, CA, USA). 

*Immunohistochemistry.* Sections were heated in a 10 mM sodium citrate pH 6.0 in a microwave to expose the antigens, and endogenous peroxidase activity was then quenched with H_2_O_2_ 0.3% in methanol. Tissue sections were incubated at 4 °C overnight with rabbit polyclonal anti-Ebp1 antibody (Abcam, Cambridge, UK) at a dilution of 1:2500. Sections were then washed and incubated with biotinylated goat anti-rabbit (Agilent Technologies, Inc., Santa Clara, CA, USA) at a dilution of 1:400. Following washing, sections were incubated with avidin–biotin complex for 30 min using the Vectastain Elite ABC kit (Vector Laboratories Inc., Burlingame, CA, USA). Following color development with 3,3-diaminobenzidine and hydrogen peroxide, sections were counterstained with hematoxylin. As a negative control, serial sections were immunostained without being exposed to the primary antibody. Finally, stained sections were examined and photographed using a Leica MZ12 dissection and a Nikon ECLIPSE Ni microscope. All images were processed in Adobe Photoshop 10.0.

*RNA interference-mediated gene silencing.* J82 cell line was transfected with specific siRNAs for the PA2G4P4 pseudogene (Silencer Select n271859, si-P4) and the Silencer Select negative control No. 1 provided from Thermo Fisher Scientific, Inc. Briefly, cells were plated at 1.7 × 10^5^ cells/well in 6-well plates and incubated for 12 h before transfection with Lipofectamine 2000 (Thermo Fisher Scientific, Inc.) using 45 nM of sequence-specific and ctlr (si-ctrl) siRNAs. Cells were then collected 24, 48, and 72 h after transfection for subsequent analysis. The transfection efficiency reached 64% inhibition of PA2G4P4 transcript quantified by RT-qPCR (Figure S2A)

*Western blot analysis.* Western blot analysis was carried out following protein cell extraction by RIPA buffer (50 mM Tris-HCl, pH 7.6, 150 mM NaCl, 1 mM MgCl_2_, 0.1% NP-40). Protein concentration was measured by the Bradford Reagent Spectrophotometer Assay (Bio-Rad Laboratories, Inc.). A total of 20 μg of protein was loaded in each lane of 6–10% acrylamide gel for SDS-PAGE. The gel was blotted on an Immobilon-P PVDF membrane (Millipore) and then treated with 5% non-fat milk (Bio-Rad Laboratories, Inc.) blocking reagent for 60 minutes at room temperature. The primary antibodies were: Anti-Ebp1 rabbit polyclonal NT antibody (No. ABE043 Merck Millipore, Italy) used to detect the p48 isoform of the EBP1 protein, and anti-GAPDH mouse monoclonal antibody (G8795 Sigma-Aldrich, actually Merck) used for normalization. The incubation with anti-EBP1 was performed overnight at 4 °C and with the anti-GAPDH for 1 h at room temperature. 

The secondary antibodies were: Goat Anti-Rabbit IgG Antibody, HRP conjugate (cat. AQ132P Sigma-Aldrich, actually Merck) to reveal EBP1 protein, and Goat Anti-Mouse IgG Antibody, HRP conjugate (cat. GtxMu003DHRPx Immunoreagent), to reveal GAPDH protein. Incubation with secondary antibodies were performed for 1 h at room temperature. All membranes were developed using the ECL kit (cat. EMP001005 Euroclone) and exposed to x-ray film (Aurogene).

*Cell proliferation assay.* To measure the cell growth rate, J82 cells silenced by si-P4 were harvested with trypsin every 24 h for 3 days and counted using a Bürker chamber. Dead cells were excluded using trypan blue staining. 

*Cell cycle assay.* J82 cells transfected with si-P4 were harvested after 48 and 72 h, fixed and permeabilized with 1 mL 70% cold ethanol. Fixed cells were then stained with a buffer containing phosphate-buffered saline (PBS), 50 μg/mL propidium iodide (Merck KGaA, Darmstadt, Germany) and 200 μg/mL RNase A (Merck KGaA). Cell cycle phase distribution was assessed using a FACS CANTO II flow cytometer (BD Biosciences, Tokyo, Japan). Data acquisition (10,000 events were collected for each sample) was performed using BD FACSDiva™ software (BD Biosciences, San Jose, CA, USA), according to the manufacturer’s instructions. Each experiment was performed in triplicate.

*Apoptotic assay.* Apoptotic cells were identified at 48 and 72 h following transfection by double staining with the Annexin V-FITC kit (Miltenyi Biotec GmbH, Bergisch Gladbach, Germany), according to the manufacturers’ instructions.

*In vitro migration assay*. Briefly, 1.7 × 10^5^ cells/well, seeded in 6-well plates, were incubated for ~36 h at 37 °C, allowing cells to completely adhere to and spread on the substrate. A single scratch was made in the cell monolayer using a micropipette tip. Subsequently, cells were washed once with PBS and further incubated, as previously described [15]. During the assay, cells were visualized using a Leica phase-contrast microscope with a 10x objective lens, and photographs of fixed positions on the wounds were captured after 0 and 24 h. The wound width was calculated by measuring the mean distance between the margins of the wound, at time points t0 (time of wounding) and t24 (24h after wounding) in randomly selected fields on the photographs. Multiple photographs of the same spots in the wound area were then captured 24h after siRNA transfection for comparison. Three independent wound-healing assays were performed.

*Statistical analysis.* All results shown are the mean of at least three independent experiments. The statistical analysis was performed using the Student’s *t*-test, except for datasets in Figure S2A,B in which a Mann–Whitney U test (*** *p* < 0.001, ** *p* < 0.005, * *p* < 0.05) was used. 

## 3. Results

### 3.1. Genomic Features of PA2G4P4 Pseudogene

**Figure 1:**
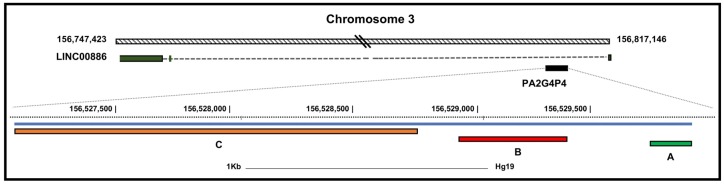
PA2G4P4 genomic location and structure. In the upper part of the figure, the relative genomic position and organization of PA2G4P4 and LINC00886 are schematically represented. In the lower part, the PA2G4P4 region is expanded. The gray line schematically represents the annotated PA2G4P4 transcript. Segment A corresponds to the 5′ RACE product, extending the PA2G4P4 region with 104 nucleotides; segment B corresponds to the in situ hybridization probe sequence; segment C spans the PA2G4 homolog region (modified from UCSC BLAT). PA2G4P4, proliferation-associated 2G4 pseudogene 4.

The *PA2G4P4* pseudogene is one of the six pseudogenes (on the 6, 9, 18, 20, and X chromosomes) of the *PA2G4* gene (NM_006191). *PA2G4P4* maps at the 3q25.31(hg38 chr3:156809551-156810732) in the intron 1 of the LINC 00,886 (NR_038387). The *PA2G4P4* processed pseudogene sequence reported in the GenBank database (NR_003284) is 2751 bp-long (Figure 1). A region of~1600 bp, corresponding to the 3′ portion of the pseudogene transcript (from 1120 to 2751) shows a similarity of ~97% with the 3′ region of the PA2G4 parental transcript localized on chromosome 12. Strikingly, the region of 1120 bp at the 5′ region of the transcript does not show any homology with the parental gene nor with any other known genomic region. According to the GTEX database (release V6), *PA2G4P4* is expressed at a very low level in a wide range of adjacent normal tissues including the bladder. 

The *PA2G4P4* transcript was further characterized by performing a 5′ RACE experiment which allowed us to extend the 5′ terminus of the transcript at 102 bp upstream of the sequence annotated in the GenBank. The non-homolog region of this non coding transcript was extended. The newly added region did not show any new significant genomic feature.

### 3.2. PA2G4P4 Expression in BlCa Samples 

**Figure 2:**
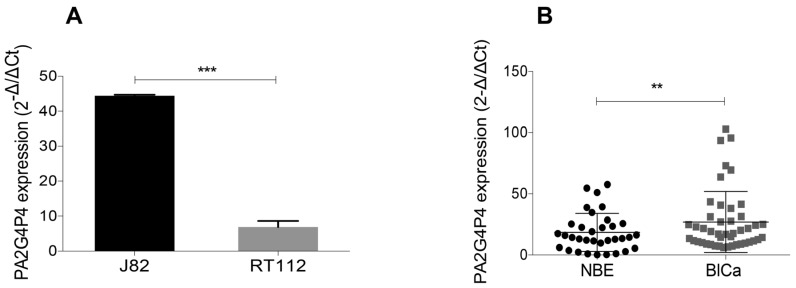
PA2G4P4 expression in BlCa cell lines and tissues. (**A**) PA2G4P4 expression level measured by RT-qPCR in J82 and RT112 cells (*** *p* < 0.001). (**B**) PA2G4P4 expression in BlCa patients (n = 45) compared to NBE samples (n = 34) (* *p* < 0.05). Data are representative of three indipendent experiments. P-values were obtained in panel (**A**) using the Student’s t test for independent samples and in panel (**B**) using the non-parametric Mann–Whitney U test.

*PA2G4P4* expression was measured in BlCa samples, either cultured cell lines (Figure 2A) or primary tissues (Figure 2B) by RT-qPCR. It was observed that the *PA2G4P4* expression increased 5-fold (*p* < 0.001) in J82 cells, a poorly differentiated and more aggressive BlCa cell line than RT112, with a more differentiated and less aggressive phenotype (Figure 2A). The *PA2G4P4* RNA levels were then compared between BlCa tissues and adjacent normal bladder epithelium, NBE (Figure 2B). All tissue samples (both tumoral and normal surrounding area) were histologically evaluated by two independent pathologists. A significant increase of the *PA2G4P4* transcript expression was observed in the BlCa samples (n = 45), as compared to the adjacent NBE ones (n = 34). The *PA2G4P4* expression analysis of all tumor samples, according to their grading and staging classification, is shown in the Supplementary Figure S1. In summary, our data indicate that the *PA2G4P4* transcript is enriched in bladder tissues clearly recognized as tumoral. 

On the same cohort of patients, we also analyzed the expression of the parental gene *PA2G4* (Supplementary Figure S2A) and, finally, on a lower number of subjects (BlCa = 23, NBE = 14,), we also detected the LINC 00,886 expression (Supplementary Figure S2B). In both cases, no significant difference was observed between BlCa and adjacent normal tissues, suggesting that only the *PA2G4P4* transcript level is related to bladder cancer development, whereas both the *PA2G4* and LINC 00,886 transcript level are not informative with respect to this pathology. 

### 3.3. PA2G4P4 Transcript Localization and Parental EBP1 Protein Distribution in BlCa

**Figure 3:**
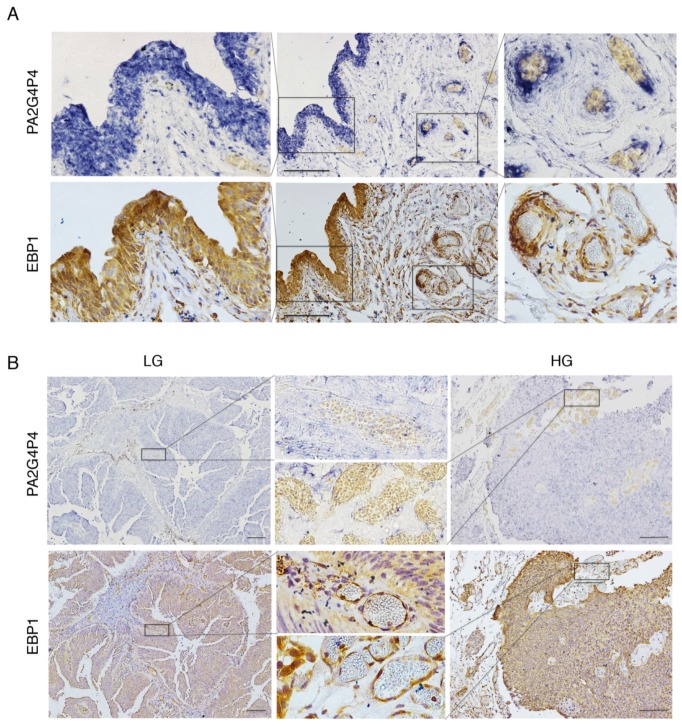
Histological analysis of the PA2G4P4 transcript and EBP1 protein. Analysis of *PA2G4P4* transcript distribution and comparison with EBP1 protein localization in both urothelial carcinomas and adjacent non-tumoral bladder tissues. Panel (**A**) refers to non-tumoral bladder tissues, while panel (**B**) shows the results on urothelial carcinomas. In both cases, serial sections were analyzed by digoxigenin in situ hybridization for *PA2G4P4* transcript detection (upper part of the panels) or EBP1 immunohistochemistry (lower part of the panels). Squares indicate the magnified areas. Bars represent 200 μm. PA2G4P4, proliferation-associated 2G4 pseudogene 4; EBP1, ErbB3-binding protein 1; LG, low-grade; HG, high-grade.

To study the *PA2G4P4* RNA localization in BICa samples, in situ hybridization was performed using a digoxigenin-labelled *PA2G4P4* RNA antisense probe (Figure 1) on both tumor samples, divided into low( LG) and high (HG) grade BlCa samples, and on adjacent bladder tissues (Figure 3). A strong positivity for *PA2G4P4* RNA was identified in the urothelium and in correspondence to the blood vessels of adjacent bladder samples (Figure 3A, upper panels). In addition, a broad *PA2G4P4* transcript distribution was found in all of the urothelial carcinomas analyzed for both LG and HG, while blood vessels in both types were negative (Figure 3B, upper panels). *PA2G4P4* RNA localization was also compared with EBP1 protein distribution by immunohistochemistry on the corresponding serial sample sections. The localization of *PA2G4P4* RNA was almost overlapping with the immunostaining for EBP1 in both normal tissues (Figure 3A, lower panels) and in the BlCa tumors (Figure 3B, lower panels), with the only exception of tumor blood vessels being immunoreactive for EBP1 (Figure 3B, lower panel), but negative for the presence of *PA2G4P4* transcripts (Figure 3B, upper panel).

All together, these data showed that *PA2G4P4* transcripts are localized in the urothelium compartment of both BlCa samples and adjacent bladder tissues, suggesting a specific role of the transcript in this type of cell. In addition, its distribution strongly correlates with EBP1 protein localization, supporting the hypothesis of a common regulation in bladder epithelium.

### 3.4. PA2G4P4 Silencing Affects Cellular Proliferation 

In order to investigate the role of PA2G4P4 in BlCa, the transcript was depleted by specific siRNA transfection (si-P4) in the J82 cell line. The siRNA sequence was selected by the Thermo Fischer Company in the non-homologous region of the *PA2G4P4* transcript to obtain a pseudogene-specific silencing. The cells depleted of pseudogene were harvested at 24, 48, and 72 h, and the PA2G4P4 RNA quantified by qPCR. The efficiency of RNA depletion was 10%, 55%, and 64%, respectively (Supplementary Figure S3A). Furthermore, the pseudogene depletion did not affect the expression of the p48 isoform of the EBP1 protein (Supplementary Figure S3B). 

**Figure 4:**
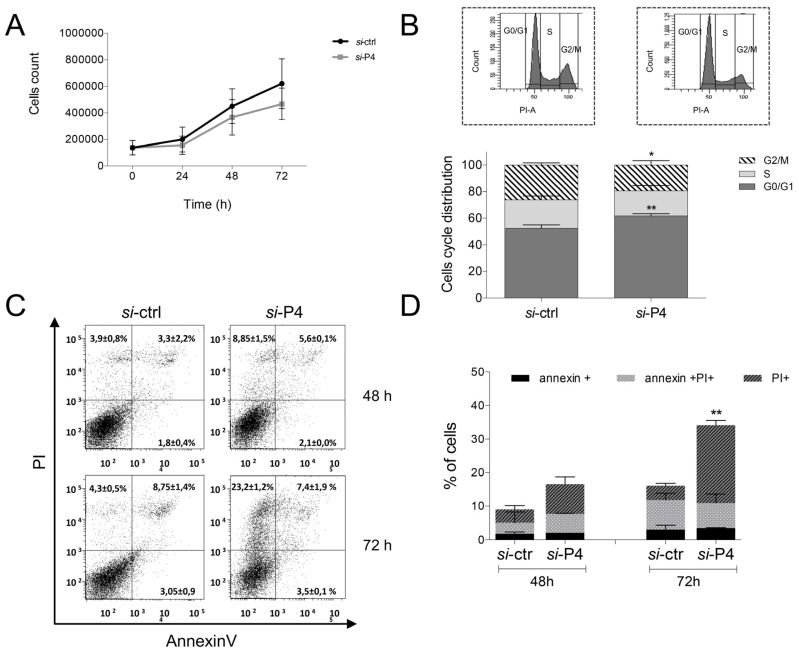
Proliferation phenotype of J82 bladder cells following PA2G4P4 silencing. (**A**) Growth rates evaluated at different time points counted using a Bürker chamber and trypan blue staining. (**B**) Analysis of cell cycle distribution in the G0/G1, S, and G2/M phases. The upper panel represents the mean of three independent experiments; the lower panel shows flow cytometry histograms of a single representative experiment (*** *p* < 0.001,* *p* < 0.05). (**C**) Analysis of apoptosis following Annexin V and PI staining. The dot plots show the percentage of cell distribution in early apoptosis (positive for Annexin V staining), late apoptosis (double positive for both Annexin V and PI staining), and necrotic cells (single positive for PI staining). PA2G4P4, proliferation-associated 2G4 pseudogene 4. (**D**) Summary graph of three independent experiments of apoptosis analysis (** *p* < 0.005).

To analyze the phenotype resulting from the pseudogene depletion, we first compared the proliferation rate of si-P4-silenced J82 cells to the control (si-ctrl). Cells were counted every 24 h, and as shown in Figure 4A, *PA2G4P4* silencing led to the decreased proliferation of BlCa cells, as compared to the control. In order to clarify the genesis of this observed phenotype, the variation of cell cycle distribution following *PA2G4P4* depletion was evaluated by flow cytometry. As shown in Figure 4B, a significant (p < 0.001) increase of the G1 phase (62%) was observed in silenced cells, as compared to the control (52%). This increase was associated with a reduction of the G2/M phase (19 vs. 26%; *p* < 0.05). These data clearly indicated that following *PA2G4P4* knockdown, J82 cells were retained in the G1 phase. Consequently, a reduction in DNA synthesis and S phase increment was assessed.

Since the permanence of cells in the G1 phase can cause apoptosis, an annexin V/PI staining was performed on silenced cells. It was observed that, 72 h following silencing, the percentage of PI^+^ cells increased from 4% in the control cell cohort to 23% in the cancer one (P < 0.001; Figure 4C). Panel D reports a summary graph of the apoptosis analysis resulting from three independent experiments, demonstrating that *PA2G4P4* depletion is able to induce apoptosis in a bladder tumor cell line.

### 3.5. PA2G4P4 Silencing Inhibits Cell Migration

**Figure 5:**
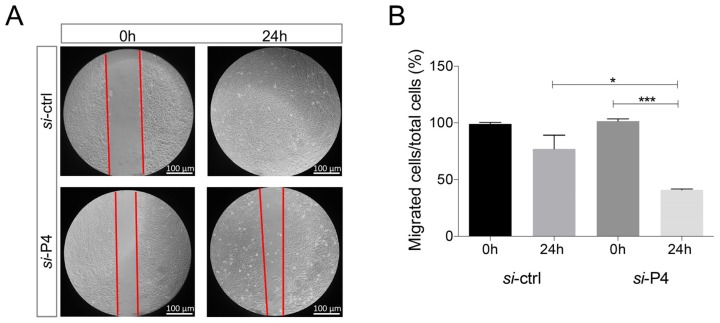
PA2G4P4 silencing effects on J82 bladder cancer migration. (**A**) Representative views of the wound healing assay were captured at 0 and 24 h, demonstrating a reduced migration of J82 cells following pseudogene silencing, as compared to the control. The scale bar in the image is 100 µm. Magnification 10X. (**B**) Quantification of cell migration by measuring the distance between the invading front of the cells in three randomly selected microscopic fields (magnification, x20) for each condition and time point. The degree of motility is expressed as the percentage of wound closure, as compared with the 0-time point. PA2G4P4, proliferation-associated 2G4 pseudogene 4. (*** *p* < 0.001,* *p* < 0.05).

An in vitro wound-healing assay was performed 36 h after *PA2G4P4* silencing (Figure 5). Imaging of cell migration revealed that pseudogene depletion decreased the motility of J82 cells in vitro by ~60% when compared with si-ctrl transfected cells (p < 0.001). This result clearly demonstrated that PA2G4P4 pseudogene-depleted cells took a longer time to fill the wound area, indicating a deficiency of J82 cells to migrate in vitro.

## 4. Discussion

In the human genome, the ENCODE project recently annotated ~15,000 human pseudogenes [20]. A growing body of evidence has suggested that individual pseudogenes are functionally involved in tumorigenesis and that can characterize tumor heterogeneity [4]. The present study focused for the first time on the *PA2G4P4* pseudogene, which has been recently identified as the most frequently appearing pseudogene in a pan-cancer classification, using RNA-seq gene expression data from the Cancer Genome Atlas [9]. We first carried out 5′ RACE experiments that allowed us to extend and define the 5′ end of the non-homolog part of the transcript. This newly identified genomic sequence is non-coding, while the 3′ end of PA2G4P4 was very difficult to delimit, as this region shows high sequence similarity with five more pseudogenes associated with EBP1 RNA as well as with several coding and non-coding EBP1 transcripts. 

Next, we measured the expression level of PA2G4P4 in two different BlCa cell lines and noticed a significant up regulation in the more differentiated one, the J82 cell line. Most importantly, when we analyzed the pseudogene expression in surgically excised bladder tumors and compared them to the surrounding normal bladder tissues, we observed a significant up regulation of the *PA2G4P4* transcript. Even though the absence of malignancy in normal bladder tissues adjacent to the tumor was histologically evaluated by two independent pathologists, we cannot exclude that the low *PA2G4P4* RNA levels detected might also be due to the presence of undetected tumoral cells into macroscopically normal tissue. However, with this being a pilot study, the small number of enrolled patients prevented us from better stratifying patients according to their TNM classification. Of note, no increase was observed in the level of the LINC 00,886 transcript overlapping PA2G4P4 at the genomic level, nor in the expression of the PA2G4 parental gene. These results suggested a specific involvement of the *PA2G4P4* pseudogene in bladder tumorigenesis. In contrast, the analysis of the Cancer Genome Atlas RNAseq dataset reported that the expression level of *PA2G4* positively correlated with that of the pseudogene, as they were both up regulated in most of the tumors analyzed including BlCas [9]. No correlation was observed in the expression levels of PA2G4P4 and PA2G4 in BlCa specimens. This divergence may have been due to the different sensitivity of the analytical methods (meta-analysis of RNA-seq datasets vs. gene specific qPCR experiments) and the complex pool of transcripts expressed by the PA2G4 locus. In support of the complexity of EBP1 detection, the mRNA and protein expression of PA2G4 was also found to be down regulated in BlCa, indicating a tumor suppressor function of PA2G4/EBP1 [14]. However, the literature contains controversial data regarding the function of EBP1 in cancer. Two isoforms of EBP1, p48 and p42, have been characterized so far, with opposite roles in tumorigenesis [21]. The major p48 isoform is considered oncogenic [22] and the p42 functions as an oncosuppressor [23]. Moreover, *PA2G4P4* transcript distribution was compared with the immunolocalization of the p48 isoform of EBP1 by analyzing serial sections of histological samples. To our knowledge, this is one of the few analyses in which pseudogene RNA and parental protein localization have been compared on serial sections of histological samples. It was found that both were localized in the urothelium of bladder tumors and adjacent non-tumoral bladder tissues, but not in the endothelium of vessels, where only the protein was detected; this suggests a specific pseudogene expression in urothelial cells. The present data highlighted the existence of a spatial correlation between *PA2G4P4* and its parental gene expression, since the two expression domains highly overlapped. The co-expression in the same cells of both *PA2G4P4* and its parental gene suggests a fine tuning of their expression and a possible synergic role in bladder cancer. In fact, due to its features, pseudogenes can essentially act as regulators of the expression of their functional counterparts [24]. The pseudogene might also control the switch among the different PA2G4 splicing variants and consequently the different protein isoforms, shown to have antithetic roles during tumorigenesis. However, the finding in the PA2G4P4 transcript sequence of a 5’ region of about 1 kb with no homology with its parental gene does not let us exclude the existence of alternative mechanisms that do not directly involve regulation of PA2G4 expression.

Finally, in order to determine the functional significance of the *PA2G4P4* pseudogene during bladder tumorigenesis, a knockdown experiment was performed in J82 cell lines by means of specific siRNA, without affecting the expression of the EBP1 protein. Phenotypic analysis showed a decreased proliferation of silenced cells and an increase in the number of cells in the G1 phase of the cell cycle. We also found an increase of apoptosis and a strong impairment of cell motility in J82 silenced cells, as compared to the control. All these data unmasked for the first time a functional role of the *PA2G4P4* pseudogene in BlCa, demonstrating that it is able to act as an oncogene essential for the maintenance of tumor cell features. 

## 5. Conclusions

Increasing evidence from large transcriptomic studies have shown that most pseudogenes are transcriptionally active and involved in important regulatory mechanisms in cancer development and progression. Our data showed, for the first time, the relevance of the *PA2G4P4* pseudogene in BlCa development, highlighting potential new implications for the diagnosis and therapeutic treatment of BlCa. Our data open to further investigation aimed to dissect the mechanism of action of the *PA2G4P4* pseudogene in BlCa and evaluate its potential role as a putative biomarker and/or therapeutic target. 

## Figures and Tables

**Table 1:** Clinical and histopathological features of bladder cancer patients.

Age at Diagnosis	Sex	Grade	Grading Stratification	Staging Stratification
<60 years (n = 10)	Male (n = 31)	Low (n = 29)	G1 (n = 26)	Ta (n = 26)
≥60 years (n = 35)	Female (n = 14)	High (n = 16)	G2–G3 (n = 19)	T1–T2 (n = 10)
				T3–T4 (n = 9)

**Table 2:** List of RACE and RT-qPCR primers.

Name.	Sequence	Genomic Position
P4Race_R1	GCCTAGGGATTAGAATGGGAGGTTA	Chr3: 156,811,797–156811821
P4Race_R2	AGTAAGAGAAACAGGGAGGGCCTAG	Chr3: 156,811,778–156811802
P4Race_R3	TGCTACATTTATTGTTTCAGGTGGG	Chr3: 156,811,955–156811979
PA2G4P4_F	CGGCTCAGGGGAAACGAGAT	Chr3: 156,810,754–156810773
PA2G4P4_R	CTCAGTACCGACACACCTGAGCT	Chr3: 156810592–156810614
PA2G4_F	AGCTCAGGTGTGTCGGTACT	Chr12: 56,106,617–56106636
PA2G4_R	GGTCGCTCTTCAAAGGGGAG	Chr12: 56,107,039–56107058
PA2G4P4insitu_F	CAGCACAGGATTCTGTTGGA	Chr3: 156,811,551–156811570
PA2G4P4insitu_R	ACAGCCTCAAAAGGCACAAT	Chr3: 156,811,125–156811144
lnc00886_F	ATGCGCATGAGAGTCATGGT	Chr3: 156,468,882–156468901
lnc00886_R	TCCACAGCAATTCACAGGCT	Chr3: 156,468,110–156468129

## Supplementary Materials

The following are available online at https://www.mdpi.com/2079-7737/9/4/66/s1, Figure S1: PA2G4P4 expression in BlCa tissues. PA2G4P4 expression level in NBE samples and BlCa according to their grading (A) and staging (B) stratification. *p*-values were obtained using the non-parametric Mann–Whitney U test (* *p* < 0.05). Figure S2: PA2G4 and LINC00886 expression in BlCa tissues. (A) PA2G4 and (B) LINC00886 mRNA quantification assessed by RT-qPCR. BlCa, bladder cancer; PA2G4P4, proliferation-associated 2G4 pseudogene 4; NBE, normal bladder epithelium. Figure S3: PA2G4P4 silencing in J82 cell line. (A) Analysis of PA2G4P4 mRNA depletion 48 and 72 h after J82 transfection with specific si-P4 (**p < 0.005). (B) Western blot performed using anti-EBP1 and anti GAPDH antibodies. The analysis was PA2G4P4, proliferation-associated 2G4 pseudogene 4; EBP1, ErbB3-binding protein 1.
