# Supplementary material for: Role of PA2G4P4 pseudogene in bladder cancer tumorigenesis
Source: Biology (Basel). 2020 Mar 31;9(4):66. doi: 10.3390/biology9040066 (PMC7235711; doi:10.3390/biology9040066)
Supplement: Supplementary file 1 [file biology-09-00066-s001.pdf]

Supplementary Materials

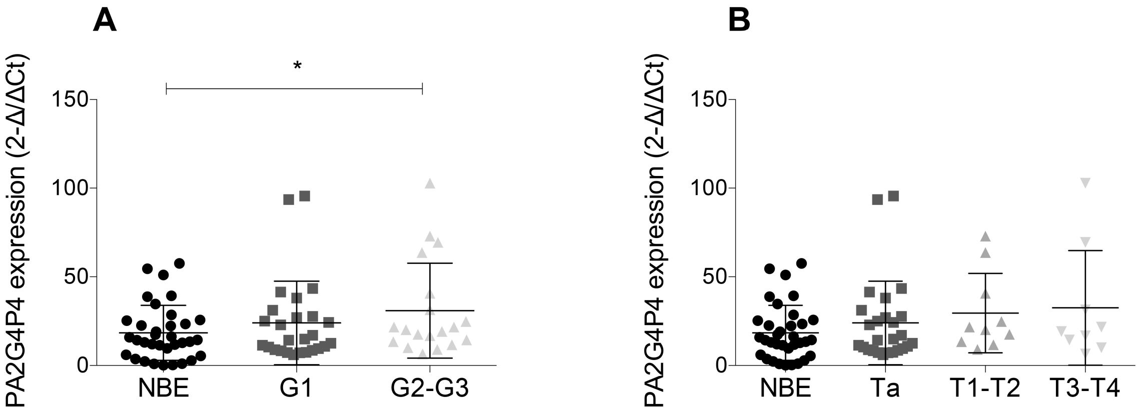

Figure S1. PA2G4P4 expression in BlCa tissues. PA2G4P4 expression level in NBE samples and BlCa according to their grading (**A**) and staging (**B**) stratification. P-values were obtained using the non-parametric Mann–Whitney U test (* $p < 0.05$).

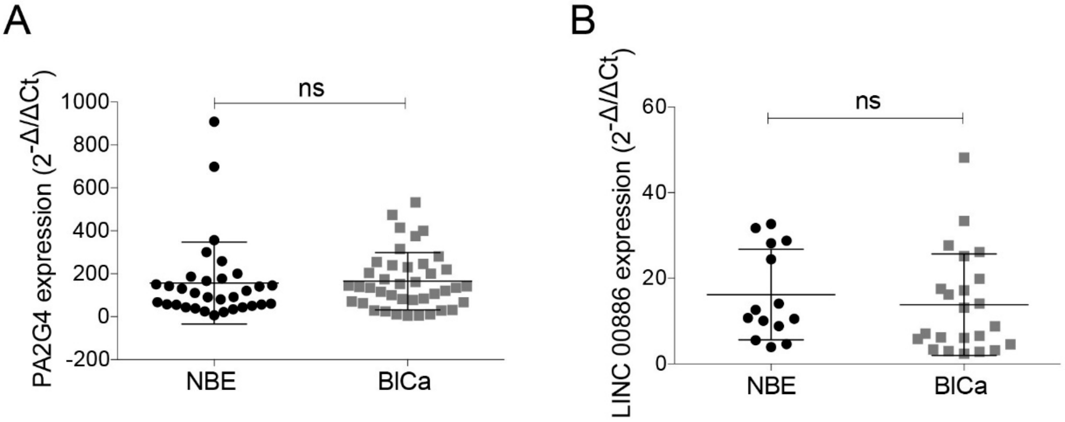

Figure S2. PA2G4 and LINC00886 expression in BlCa tissues. (**A**) PA2G4 and (**B**) LINC00886 mRNA quantification assessed by RT-qPCR. BlCa, bladder cancer; PA2G4P4, proliferation-associated 2G4 pseudogene 4; NBE, normal bladder epithelium.

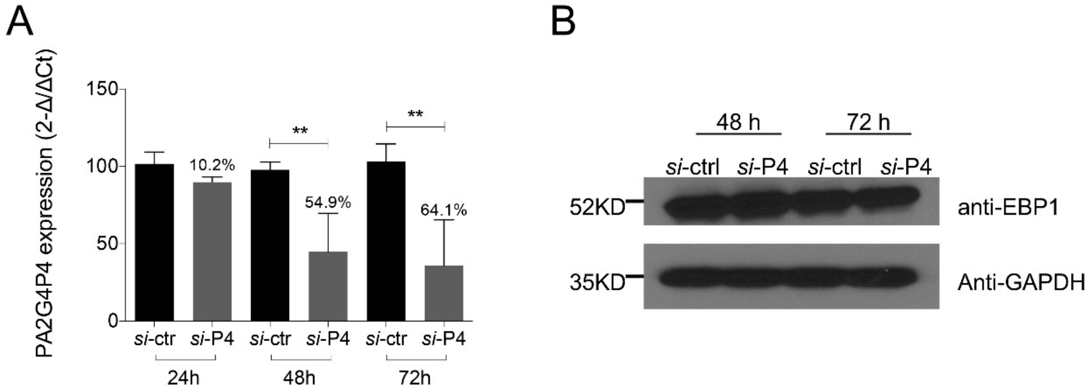

Figure S3. PA2G4P4 silencing in J82 cell line. (**A**) Analysis of PA2G4P4 mRNA depletion 48 and 72 h after J82 transfection with specific si-P4 (**$p < 0.005$). (**B**) Western blot performed using anti-EBP1 and anti GAPDH antibodies. The analysis was PA2G4P4, proliferation-associated 2G4 pseudogene 4; EBP1, ErbB3-binding protein 1.
